# Patient derived mutation W257G of *PPP2R1A* enhances cancer cell migration through SRC-JNK-c-Jun pathway

**DOI:** 10.1038/srep27391

**Published:** 2016-06-07

**Authors:** Ae Lee Jeong, Sora Han, Sunyi Lee, Jeong Su Park, Yiling Lu, Shuangxing Yu, Jane Li, Kyung-Hee Chun, Gordon B. Mills, Young Yang

**Affiliations:** 1 Department of Biological Sciences, Sookmyung Women’s University, Seoul 140-742, Republic of Korea; 2 Department of Systems Biology, The University of Texas MD Anderson Cancer Center, Houston, Texas 77030, USA; 3 Department of Biochemistry and Molecular Biology, Yonsei University College of Medicine, 50 Yonsei-ro, Seodaemun-gu, Seoul 120–752, Republic of Korea

## Abstract

Mutation of *PPP2R1A* has been observed at high frequency in endometrial serous carcinomas but at low frequency in ovarian clear cell carcinoma. However, the biological role of mutation of PPP2R1A in ovarian and endometrial cancer progression remains unclear. In this study, we found that *PPP2R1A* expression is elevated in high-grade primary tumor patients with papillary serous tumors of the ovary. To determine whether increased levels or mutation of PPP2R1A might contribute to cancer progression, the effects of overexpression or mutation of PPP2R1A on cell proliferation, migration, and PP2A phosphatase activity were investigated using ovarian and endometrial cancer cell lines. Among the mutations, PPP2R1A-W257G enhanced cell migration *in vitro* through activating SRC-JNK-c-Jun pathway. Overexpression of wild type (WT) PPP2R1A increased its binding ability with B56 regulatory subunits, whereas PPP2R1A-mutations lost the ability to bind to most B56 subunits except B56δ. Total PP2A activity and PPP2R1A-associated PP2Ac activity were significantly increased in cells overexpressing PPP2R1A-WT. In addition, overexpression of PPP2R1A-WT increased cell proliferation *in vitro* and tumor growth *in vivo.*

Endometrial carcinoma, a heterogeneous disease, is the most frequently diagnosed gynecological cancer. Endometrial carcinoma is traditionally classified into two main groups: type Ι (low-grade endometrioid carcinoma), and type ΙΙ (mainly endometrial serous carcinoma)^1^. Although type ΙΙ endometrial carcinomas constitute only 10% of all endometrial cancers, they account for a disproportionately high number of deaths due to their unique tendency to metastasize^2^. Through genome-wide analyses, molecular genetic aberrations involving p53, cyclin E-FBXW7, ARID1A, and PI3K pathways have been uncovered as major factors involved in the development of endometrial serous carcinoma^3^. In addition to endometrial carcinoma, ovarian cancer, one of the most lethal gynecologic malignancies, is the fifth leading cause of cancer-related death among women^4^. Ovarian cancer is also a heterogeneous disease with distinct genetic patterns and metastasis to the peritoneal cavity during advanced stage with poor prognosis^5^,^6^. To identify driver mutations for ovarian cancer, many studies have employed comprehensive exome sequencing to search for somatic mutations in primary tumors. *PPP2R1A* coding a scaffold subunit of protein phosphatase 2A (PP2A) has been found to be one recurrent mutation in both ovarian and endometrial cancers^3^,^7^,^8^,^9^,^10^.

PP2A, one of four major serine/threonine phosphatases, is a heterotrimeric phosphatase containing a scaffold subunit (PR65), a catalytic subunit (PP2Ac), and a B regulatory subunit^11^. PP2A regulates a variety of cellular functions, including cell cycle regulation, mitosis, and DNA damage repair through a broad spectrum of substrates^12^,^13^,^14^. Moreover, PP2A is predominantly regarded as a tumor suppressor. Restoration of PP2A activity benefits some cancer patients^15^,^16^. However, some studies have shown that PP2A may have opposite effect on tumor progression^17^,^18^,^19^,^20^,^21^. PR65 serves as a scaffold to coordinate the interaction between the core enzyme and a wide range of B regulatory subunits, allowing specific temporal targeting of substrates to PP2A. PR65 has two isoforms (PPP2R1A and PPP2R1B) that share 86% amino acid sequence identities^22^. Each isoform contains 15 huntingtin-elongation-A subunit-TOR (HEAT) repeats. Of the 15 repeats, repeats 1–10 bind to B regulatory subunit whereas repeats 11–15 bind to PP2Ac^23^. PPP2R1A can bind to T antigen (TAg) from both SV40 and polyoma virus, thus providing evidence that *PPP2R1A* can function as cancer relevant genes. *PPP2R1A* somatic mutations (E64D, E64G, and R418W) have been identified in lung carcinoma, breast carcinoma, and melanoma^24^. These mutations contribute to human cell transformation by disrupting the composition of PP2A complex and reducing phosphatase activity^25^. *PPP2R1B* missense mutations (P65S, L101P, K343E, D504G, and V545A) and homozygous deletions detected in lung and colon cancers^26^ contribute to the loss of PP2Ac binding^27^.

Recently, mutations in *PPP2R1A* have been identified at low frequency in ovarian clear cell carcinoma^28^. Subsequent studies have revealed that *PPP2R1A* mutations occur at high frequency in endometrial serous carcinomas^7^,^29^,^30^. These recurrent *PPP2R1A* mutation sites are mainly located within the TAg binding site for polyoma virus and SV40. However, the biological role of these mutations of PPP2R1A in ovarian and endometrial cancer progression remains unclear. Therefore, the objective of this study was to determine whether *PPP2R1A* mutations contribute to cancer progression through affecting cell proliferation, migration, and PP2A phosphatase activity.

## Results

### *PPP2R1A* mutations at the TAg binding site are recurrent in human endometrial and ovarian cancers

Oncomine database was used for analyzing *PPP2R1A* expression level across human cancer types using methods described previously^31^. The expression levels of *PPP2R1A* are significantly increased in high-grade ovarian serous carcinoma, ovarian serous adenocarcinoma, invasive breast carcinoma, melanoma, lung adenocarcinoma, and bladder carcinoma compared to those in normal tissues^32^,^33^,^34^,^35^,^36^,^37^,^38^,^39^,^40^ (Table 1). For ovarian cancers, there are seven different studies, including those of Bonome, T. *et al.*^33^, Lu, K. H. *et al.*^34^, Welsh, J. B. *et al.*^41^, Adib, T. R. *et al.*^42^, Yoshihara, K. *et al.*^43^, Hendrix, N. D. *et al.*^44^, and TCGA. Among the seven studies, *PPP2R1A* mRNA expression level is clearly increased in stage III high-grade papillary serous ovarian tumors in the study of Bonome, T. *et al.*. However, *PPP2R1A* mRNA expression level is not elevated in various stages of ovarian serous cystadenocarcinoma in the TCGA database, indicating that *PPP2R1A* expression is only increased in high-grade carcinomas. In addition to overexpression of *PPP2R1A*, *PPP2R1A* mutations have been found in different cancer types (Table 2), including ovarian carcinoma, endometrial carcinoma, breast invasive carcinoma, colorectal adenocarcinoma, lung adenocarcinoma, and renal clear cell carcinoma based on cBio Portal for Cancer Genomics (www.cbioportal.org).

**Figure 1:**
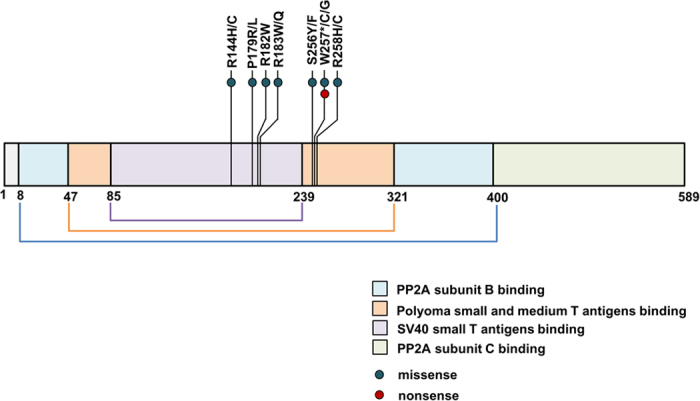
Schematic diagram showing structural domains of PPP2R1A protein (589 amino acids). The positions of recurrent mutations found in ovary and endometrial carcinomas are marked. Different domains are highlighted with different colors as indicated at the bottom of the figure.

Among different *PPP2R1A* mutations, P179R, R183W, S256F, and W257G mutation were selected for functional analysis because P179R and R183W were located at polyoma small T antigen binding site of PPP2R1A while S256F and W257G were located at SV40 small T antigen binding site (Fig. 1). Their mutation rates in various ovarian and endometrial carcinomas studies are summarized in Table 3.

### Overexpression of PPP2R1A-WT promotes cell proliferation and PPP2R1A-W257G increases cell migration of ovarian cancer cells

**Figure 2:**
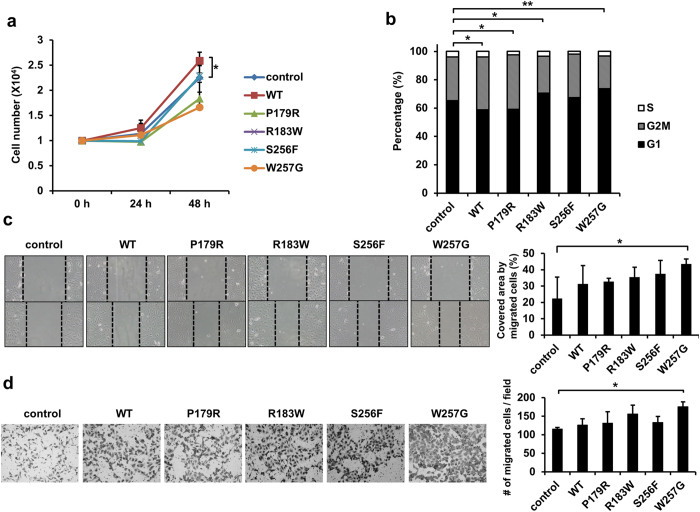
Overexpression of PPP2R1A-WT promotes cell proliferation and overexpression of PPP2R1A-W257G increases cell migration of SKOV3 cells. (**a**) Control, PPP2R1A-WT, P179R, R183W, S256F, or W257G SKOV3 cells (1 × 10^4^) were plated into 48-well plates. These cells were counted at indicated times. Results from three independent experiments are shown. *p < 0.05; one-tailed Student’s t test. (**b**) Control, WT, and mutant cells (1 × 10^6^) were plated into 100-mm culture plates. On the next day, cells were fixed and stained with propidium iodide solution for DNA staining. Flow cytometry was used to analyze DNA content. The percentages of cells in G1, G2M, and S phases of the cell cycle are indicated. Data are shown as means. n = 3. *p < 0.05; **p < 0.01; one-tailed Student’s t test (G1 phase). (**c**) Control, WT, and mutant cells (1 × 10^5^) were plated into 12-well plates. Cells were wounded by scratching the surface with a pipette tip. The images of wound-scratch assays were taken at 0 and 15 h post scratching. Relative closed-wound distance was calculated after measuring the width of at least four wounds. *p < 0.05; one-tailed Student’s t test. (**d**) Stable cells (1 × 10^4^) were plated onto the upper part of a trans-well chamber in serum-free media. The medium of the lower chamber contained 10% FBS. After incubation for 48 h, cells migrated to the lower surface of the membrane were fixed with 4% formaldehyde, stained with Giemsa, and counted under a microscope. The mean ± S.D. of measurements in triplicates are indicated. *p < 0.05; one-tailed Student’s t test.

PPP2R1A mutants were overexpressed in SKOV3 ovarian carcinoma cells to determine the effect of PPP2R1A mutations on tumor cell growth. Overexpression of PPP2R1A-WT modestly but significantly enhanced cell proliferation compared to control cells (Fig. 2a). In contrast, constructs of PPP2R1A mutants failed to increase cell proliferation. In fact, P179R and W257G mutants modestly decreased cell growth rate. Consistent with cell proliferation result, PPP2R1A-WT had the lowest (58.6%) G1 population based on cell cycle analysis while all other mutants showed higher proportions of G1 phase cells (R183W, 70.4%; S256F, 67.3%; and W257G, 73.6%) than control (65.1%) except P179R mutant which showed lower proportion of G1 phase cells (59.06%) than control cells (Fig. 2b).

To determine the effects of PPP2R1A overexpression and PPP2R1A mutations on cancer cell migration, wound-scratch assays were performed to determine the migration capability of SKOV3 cells. Interestingly, migration of SKOV3 cells overexpressed with W257G mutant of PPP2R1A was significantly increased compared to other experimental groups (Fig. 2c). Consistent with results of wound scratch assays, trans-well assay also revealed that W257G was the most effective mutation with 1.5-fold increase in the number of migrated cells compared to the control group (Fig. 2d). Taken together, these results indicate that the expression level of *PPP2R1A* is highly increased in various human carcinomas and the overexpression of the PPP2R1A-WT promotes SKOV3 cell proliferation. However, W257G mutant of PPP2R1A can effectively increase cell motility.

### PPP2R1A-WT increases cell proliferation and PPP2R1A-W257G enhances cell migration of endometrial cancer cells

**Figure 3:**
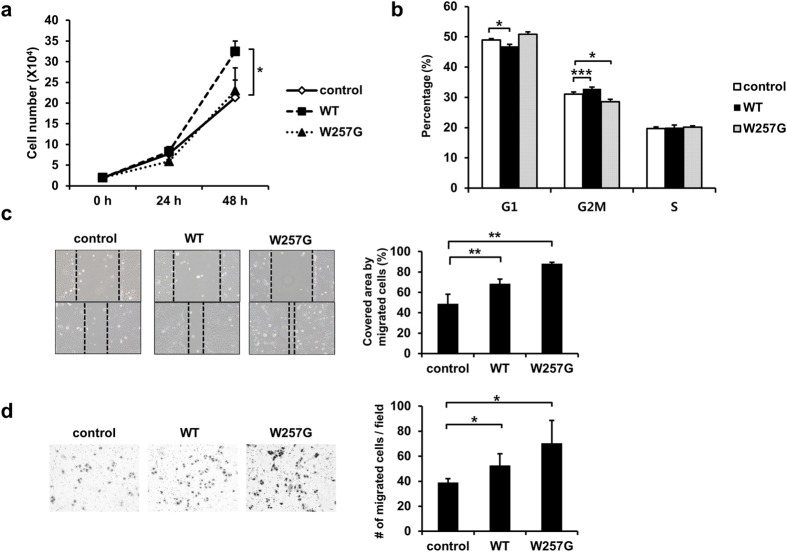
Overexpression of PPP2R1A-WT increases cell proliferation and overexpression of PPP2R1A-W257G enhances cell migration of HEC-251 cells. (**a**) Control, PPP2R1A-WT, and W257G stably expressing HEC-251 cells (2 × 10^4^) were plated into 48-well plates. These cells were counted at indicated times. Results of three independent experiments are shown. *p < 0.05; one-tailed Student’s t test. (**b**) Control, WT, and W257G cells (2 × 10^6^) were plated into 100-mm culture plates. On the next day, these cells were fixed and stained with propidium iodide solution for DNA staining. Flow cytometry was used to analyze DNA content. The percentages of cells in G1, G2M, and S phases of the cell cycle are indicated. Data are shown as means. n = 3. *p < 0.05, ***p < 0.001; one-tailed Student’s t test (G1 phase). (**c**) Control, WT, and W257G cells (2 × 10^5^) were plated into 12-well plates. Cells were wounded by scratching the surface with a pipette tip. The images of wound-scratch assays were taken at 0 and 15 h post scratching. The relative closed-wound distance was calculated after measuring the width of at least four wounds. **p < 0.01; one-tailed Student’s t test. (**d**) Stable cells (1 × 10^4^) were plated onto the upper part of a trans-well chamber in serum-free media. Cells migrated to the lower surface of the membrane were counted. The mean ± S.D. of measurements in triplicates are indicated. *p < 0.05; one-tailed Student’s t test.

The effects of WT and W257G mutant of PPP2R1A were examined in endometrial cancer cell line HEC-251. Consistent with results obtained in SKOV3 cells, PPP2R1A-WT significantly increased cell proliferation and decreased the proportion of G1 phase cells (46.9%) compared to HEC-251 control cells (48.9%). However, W257G had no significant effect on cell proliferation (Fig. 3a,b). Similar to results in SKOV3, HEC-251 cells expressing W257G mutant had higher cell motility (Fig. 3c,d). These data indicate that WT can increase cell proliferation and W257G mutant has an important role in cell migration in both SKOV3 and HEC-251 cancer cells.

### PPP2R1A mutations do not alter PP2A enzyme activity but alter the interaction with PP2Ac and B56 regulatory subunits

**Figure 4:**
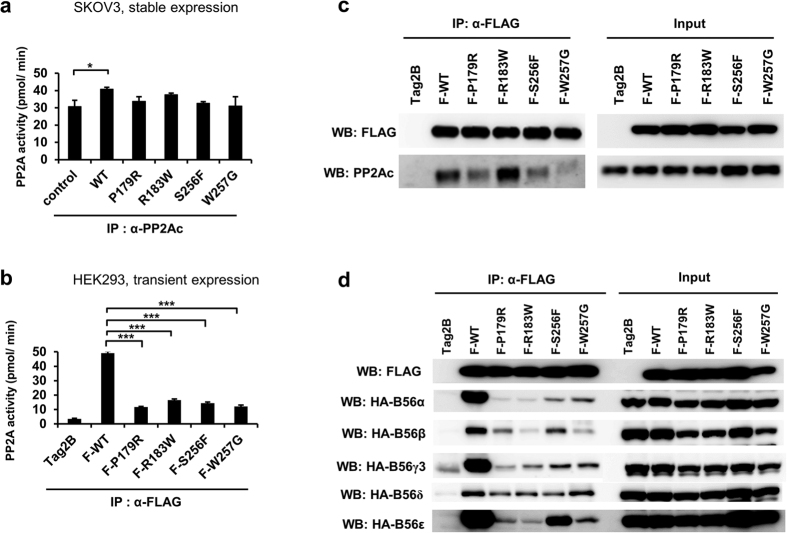
PPP2R1A mutations have no effect on PP2A enzyme activity but reduce their interactions with PP2Ac and B56 regulatory subunits. (**a**) Each lysate of SKOV3 cell stably expressing PPP2R1A was immunoprecipitated with anti-PP2Ac antibody. PP2A enzyme activity was measured by Serine/Threonine phosphatase assay system using phosphopeptide as substrate. (**b**) FLAG-PPP2R1A constructs were overexpressed in HEK293 cells. At 48 h post transfection, FLAG-WT and mutants were purified using anti-FLAG M2 affinity gel. Protein phosphatase activity was measured using purified FLAG-WT. Mutations as described in (**a**). Data are shown as mean ± S.D of three independent experiments (n = 3).*p < 0.05, ***p < 0.001; one-tailed Student’s t test. (**c**) The immunoprecipitates and whole-cell lysates (Input) were subjected to immunoblotting using anti-FLAG and anti-PP2Ac antibodies. (**d**) FLAG-PPP2R1A constructs were overexpressed with HA-B56 subunits in HEK293 cells. At 48 h post transfection, cells were lysed and immunoprecipitated with FLAG antibody. The immunoprecipitates and input were subjected to immunoblotting using anti-FLAG and anti-HA antibodies.

To determine whether altered cell proliferation and migration capability are correlated with PP2A activity, total PP2A enzyme activity was examined in cells overexpressing WT or each mutant. When each cell lysate was immunoprecipitated with an anti-PP2Ac antibody, the PP2A activity of cell lysates containing overexpressed PPP2R1A-WT was the only one that was significantly increased (approximately 13.3% increase compared to the control, Fig. 4a). PPP2R1A-associated PP2A activity was also measured to exam the specific PP2A activity of cell lysates containing overexpressed mutants. FLAG-tagged WT and each mutant (F-WT, P179R, F-183W, F-S256F, and F-W257G) were transiently expressed and immunoprecipitated using an anti-FLAG antibody to pull down PPP2R1A-associated PP2Ac. Surprisingly, immunoprecipitates from cells overexpressing mutant PPP2R1A showed very little phosphatase activity compared to F-WT (Fig. 4b). To determine whether PPP2R1A mutation-associated activity was affected by the difference in the amount of bound PP2Ac, the level of PP2Ac in each immunoprecipitate was examined. The interaction between PPP2R1A mutants and PP2Ac was impaired except R183W mutant (Fig. 4c). This result indicates that the reduced PP2A activity is likely due to the amount of PP2Ac bound to PPP2R1A. However, it remains to be determined why R183W mutant has lower phosphatase activity despite the fact that it has interaction with PP2Ac. Since the specificity of PP2A substrate is determined by B56 regulatory subunits, the binding affinity between B subunit and each mutant was assessed. The binding of B56 regulatory subunits was severely impaired in mutants except B56δ that can bind to all mutants (Fig. 4d). These results indicate that PPP2R1A mutations found in cancer patients can alter their interactions with PP2Ac and B56 regulatory subunits.

### PPP2R1A-W257G elevates the phosphorylation of SRC, JNK, and c-Jun

**Figure 5:**
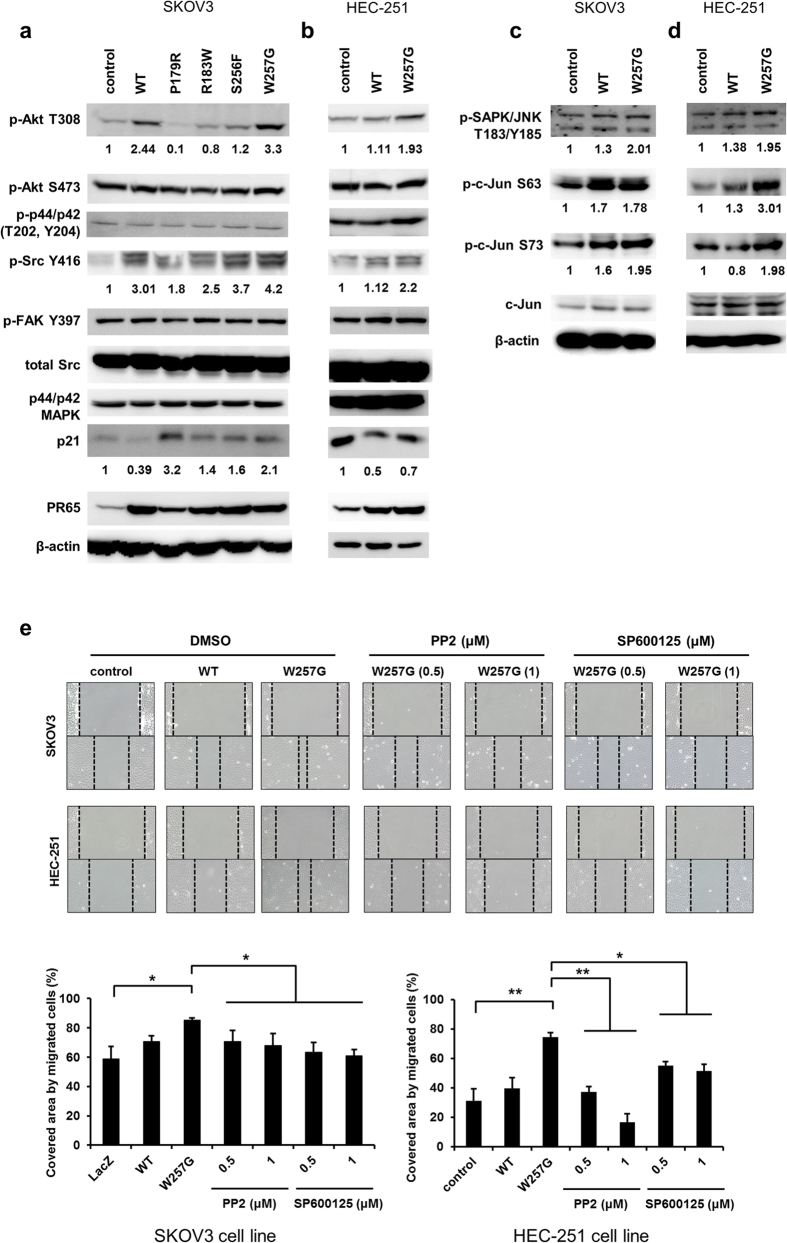
PPP2R1A-W257G elevates phosphorylation of SRC, JNK, and c-Jun. (**a–d**) SKOV3 and HEC-251 cells stably expressing PPP2R1A were serum starved for 12 h. These cells were lysed. The phosphorylation levels of AKT, p44/42, SRC, FAK, SAPK/JNK, and c-Jun and the expression levels of SRC, p44/p42, p21, PR65, b-actin, and c-Jun were analyzed by western blotting. (**e**) After cells were wounded by scratching the surface with a pipette tip, cells were treated with PP2 or SP600125 at indicated concentrations. The images of wound-scratch assays were taken at 0 and 15 h post scratching. The relative closed-wound distance was calculated after measuring the width of at least four wounds. *p < 0.05, **p < 0.01; one-tailed Student’s t test.

To explain how cell proliferation and migration are increased by WT and W257G, downstream signaling pathways in cell proliferation and migration including p21, AKT, MAPK, SRC, and FAK were examined. Consistent with the effects of WT on cell proliferation, cyclin-dependent kinase inhibitor p21^WAF1/Cip1^ levels were notably decreased to 39% in SKOV3 cells and 50% in HEC-251 cells overexpressing PPP2R1A-WT. No alteration in phosphorylation level of Ser473 in AKT, Thr202/Tyr204 in MAPK, or Tyr397 in FAK was observed. However, the phosphorylation level of Thr308 in AKT and Tyr416 in SRC were elevated in SKOV3 cells and HEC-251 endometrial cells overexpressing WT and W257G mutant of PPP2R1A (Fig. 5a,b).

Since JNK and c-Jun are downstream molecules in the SRC signaling pathway with essential roles in cell migration^45^,^46^,^47^, the phosphorylation levels of JNK and c-Jun were measured. JNK phosphorylation level was increased 1.3-fold in both SKOV3 and HEC-251 cells overexpressing the WT and increased 2-fold in these cells overexpressing the W257G mutant (Fig. 5c,d). As expected, the phosphorylation levels of c-Jun Ser63 and Ser73 were also significantly increased in both SKOV3 and HEC-251 cells overexpressing the W257G mutant but slightly increased in these cells overexpressing the WT of PPP2R1A (Fig. 5c,d). Next, we examined whether inhibition of SRC or JNK pathway could block cell migration of SKOV3 and HEC-251 cells overexpressing the W257G mutant. Our results revealed that treatment with PP2, a selective inhibitor for SRC-family kinases, reduced cell migration. Furthermore, SP600125, a selective inhibitor of JNK, also reduced the effect of W257G on migration of both SKOV3 and HEC-251 cells (Fig. 5e). These results imply that the SRC and JNK signaling pathways are required for W257G-enhanced migration.

### PPP2R1A-WT promotes tumor growth *in vivo*

**Figure 6:**
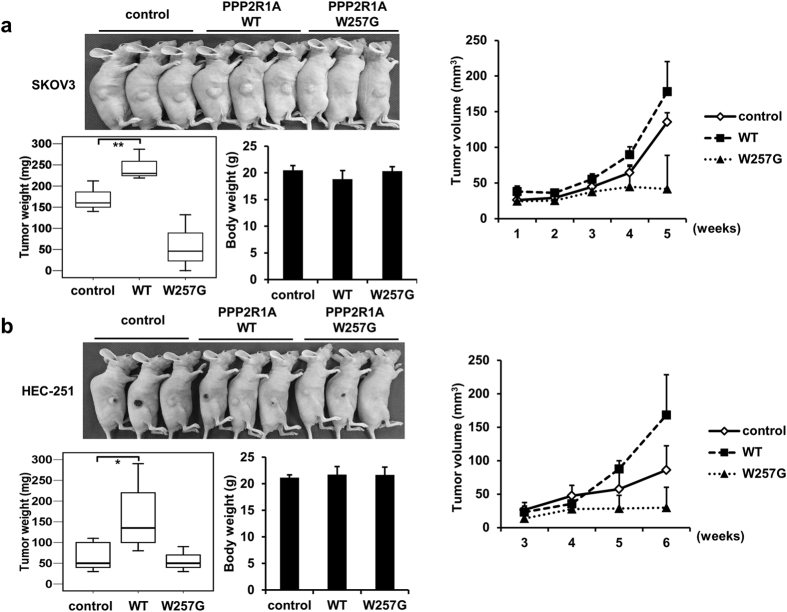
PPP2R1A-WT promotes tumor growth *in vivo*. (**a–b**) Mice were injected with 2 × 10^6^ of SKOV3 cells or 6 × 10^6^ of HEC-251 cells stably expressing PPP2R1A. Xenograft tumors, tumor weight, body weight, and tumor volume are shown. Means ± S.D. for measurements in triplicates are indicated. *p < 0.05, **p < 0.001; two-tailed ANOVA and Scheffe’s test.

Next, the effect of overexpressed PPP2R1A on tumor growth was evaluated in a xenograft model. Consistent with *in vitro* results, both SKOV3 and HEC-251 cells overexpressing PPP2R1A-WT showed a dramatic increase in tumor growth. However, both SKOV3 and HEC-251 cells overexpressing the W257G mutant showed less tumor growth compared to parental cells (Fig. 6a,b).

## Discussion

Because inhibition of PP2A activity induces neoplastic transformation, PP2A is considered as a tumor suppressor. It has been reported that the restoration of PP2A activity can benefit some cancer patients^15^,^16^. However, PP2A might also promote tumor progression. For example, PP2Ac inhibits p53-mediated apoptosis in hepatocellular cancer cells and is positively associated with the survival of leukemic, pancreatic, and glioblastoma cells^17^,^18^,^19^,^20^,^21^. In addition, PP2Ac-overexpressing mice generate many hepatocellular tumors^18^,^48^. Furthermore, increased PP2A activity also contributes to drug resistance in HER2 positive subtype of breast cancer^49^. This study provides one more piece of evidence supporting the oncogenic role of PP2A.

*PPP2R1A*-WT is upregulated in various types of cancers (Table 1). Our results revealed that overexpression of PPP2R1A-WT could enhance cell proliferation (Fig. 2a and 3a). Among mutations, W257G could increase cell migration the most (Fig. 2c,d). Thus, the ability of tumor formation was examined in xenograft models using endometrial and ovarian cancer cells overexpressing WT or W257G. Surprisingly, only cells overexpressing WT enhanced tumor formation while cells overexpressing W257G decreased tumor formation. WT-overexpressing cells showed increased PP2A activity when immunoprecipitated with anti-PP2Ac antibody (Fig. 4). The increased PP2A activity in cells overexpressing WT was found to be due to increased binding of PP2Ac with PPP2R1A-WT. However, cells overexpressing W257G did not enhance PP2A activity. They decreased tumor growth *in vivo*. Collectively, these results suggest that increased PP2A activity plays an oncogenic role. To support the idea that enhanced PP2A activity is associated with tumor formation, tumor formation ability of cells overexpressing R183W mutant needs to be examined by using xenograft model because this R183W mutant increased PP2Ac binding without increasing PP2A activity.

Because PP2Ac is associated with different scaffold and regulatory subunits, several holoenzymes of PP2Ac with distinct functions and characteristics can be produced. While PP2Ac and scaffold subunit sequences have remarkable sequence conservation, regulatory subunits are more heterogeneous, suggesting that the regulatory subunits might play key roles in controlling the localization and specific activity of different holoenzymes. The results of this present study revealed that overexpression of PPP2R1A-WT could lead to increased B56 regulatory subunit binding while PPP2R1A-mutations showed decreased binding except B56δ. Altered binding to regulatory subunits might in turn affect the functional diversity and complexity of various PP2A holoenzymes. Therefore, detailed biochemical studies using each purified subunit of PP2A are needed to reveal the exact molecular effect of each PPP2R1A mutation.

The W257G mutation increased cell migration in both ovarian and endometrial cancer cells. In addition, it activated important players in signaling pathway of cell migration including SRC, JNK, and c-Jun. Because total PP2Ac activity was maintained while W257G-associated PP2Ac activity was reduced, it might be hard to link W257G mutation to SRC/JNK activation. Nonetheless, such link is possible. First, W257G may obtain function to activate SRC without decreasing PP2Ac activity. Second, PP2A holoenzyme containing W257G may lose a B regulatory subunit binding that guides enzyme to SRC. For example, it is known that B55γ can bind to c-SRC and stimulate the dephosphorylation of serine 12 of SRC, a residue required for JNK activation by SRC^50^. If PP2A holoenzyme containing W257G decreases its interaction with B55γ, it could be an additional mechanism for SRC activation. These possibilities are currently under investigation.

The W257G mutation has been found in many *PPP2R1A* studies^3^,^7^,^10^,^51^. This mutation is relatively rare compared to P179R or R183W mutation. W257G mutation has relatively low frequency in polyoma virus’ small and medium T antigens binding region. On the other hand, P179R and R183W mutations are hot spot mutations in SV40’s small T antigens binding region. However, these two mutations have no significant effect on tumor migration or growth. The AKT/SRC signaling pathway was also observed in our experimental model. In contrast, W257G mutation upregulated the AKT/SRC signaling pathway associated with the increased migration. At this moment, it is difficult to consider that W257G is a cancer-driver mutation. However, our results cautiously suggest that W257G mutation might play a significant role in endometrial serous carcinomas. Further research study will yield more information on whether W257G mutation is correlated with clinical phenotypes (including clinical stage, overall survival, and metastasis) in cancer patients. Le Gallo, M. *et al.* have reported that all patients having P179R mutation have co-mutation of *TP53* but not *PIK3CA* mutation^7^. Shih Ie, M. *et al.* have reported that some patients having R183W/Q also have co-mutation of *KRAS* G12D/R^10^. Thus, although we failed to find the critical function of P179R and R183W mutations in our experimental system, it will be worthy to study the function of P179R and R183W mutations using proper cells that have co-mutations.

In the current study, overexpression of *PPP2R1A* mRNA was observed in advanced stage primary tumors. In addition, *PPP2R1A* mutation was associated with increased cell migration. However, it has been previously reported that W257G, S256F, and S256Y *PPP2R1A* mutations are present at both serous endometrial intraepithelial carcinoma (SEIC) and uterine serous carcinoma (USC)^3^. Because SEIC is the pre-invasive precursor of USC, indicating that mutations in *PPP2R1A* occur early during tumor progression of USC. Although *PPP2R1A* mutations are early events, the phenotypes of mutation could be buried until mutations of other genes start to unveil the phenotypes of *PPP2R1A* mutants. In fact, W257G and *TP53* mutations co-occur in SEIC with additional *FBXW7* mutation in USC^3^. Thus, additional *FBXW7* mutation might contribute to the unveiling of W257G function.

In summary, for the first time, we demonstrated that *PPP2R1A* mRNA expression level was elevated in high-grade ovarian serous carcinoma, invasive breast carcinoma, melanoma, lung adenocarcinoma, and bladder carcinoma. The overexpression of WT PPP2R1A promoted tumor growth while W257G mutation increased cell migration through the SRC-JNK-c-Jun pathway. Our results provide more evidence supporting the oncogenic role of PP2A.

## Materials and Methods

### Oncomine data mining

Datasets from the Oncomine cancer microarray database (https://www.oncomine.org/resource/main.html) were selected to determine the alterations of *PPP2R1A* mRNA expression.

### Plasmids

Human cDNA of PPP2R1A-WT and various mutations (P179R, R183W, S256F, W257G) were generated by PCR, sequence confirmed. The full length of each construct was prepared by PCR and subcloned into the EcoRΙ-XhoΙ sites of the pCMV-Tag2B mammalian expression vector. HA-tagged B56 subunits were purchased from Addgene (http://www.addgene.org).

### Antibodies and chemicals

The following antibodies were used: PR65 (sc-15355, Santa Cruz, CA, USA), PP2Ac (05-421, Millipore), FLAG M2 (F1804, Sigma Aldrich), HA (sc-7392, Santa Cruz), phospho-AKT (Thr308) (9275, Cell Signaling), phospho-AKT (Ser473) (9271, Cell Signaling), phospho-SRC (Tyr416) (6943, Cell Signaling), phospho-FAK (Tyr397) (3283, Cell Signaling), phospho-PP2Ac (Tyr307) (ab32104, Abcam), SRC (2108, Cell Signaling), p21 (2946, Cell Signaling), β-actin (sc-4778, Santa Cruz), phospho-SAPK/JNK (Thr183, Tyr185) (9251, Cell Signaling), phospho-c-Jun (Ser73) (9164, Cell Signaling), phospho-c-Jun (Ser63) (9261, Cell Signaling), and c-Jun (sc-1694, Santa Cruz).

### Cell Culture

The human ovarian cancer cell line, SKOV3, and human endometrial adenocarcinoma cell line, HEC-251, were obtained from the ATCC and the Characterized Cell line core of the MD Anderson Cancer Center (MDACC). SKOV3 and HEC-251 cells were cultured using RPMI1640 (Hyclone) supplemented with 10% FBS, and HEK293 cells were cultured using Dulbecco’s modified Eagle’s medium (Hyclone) supplemented with 10% FBS at 37 °C in a humidified atmosphere of 5% CO_2_ in air.

### Generation of stable cell lines expressing each mutant

For stable transfection, the 3^rd^ generation of the lentiviral packaging system (VSV-G, GagPol, REV, TAT) was used. Mutant constructs were cloned into a plenti6.3 vector, and viruses were produced by transfecting plasmids in HEK 293T cells. After collecting the virus, SKOV3 and HEC-251 cells were infected for two rounds. 72 hours after infection, cells were selected by blasticidin (10 μg/ml). Similar levels of each of the constructs were present in the cells (see Fig. 5a,b).

### Cell proliferation assay

For the proliferation assay, SKOV3 and HEC-251 stable cells were seeded into 48-well plates at a density of 1 × 10^4^ cells per well, respectively. The cells were counted at 24 and 48 h after plating.

### Wound scratch assay

For wound scratch assays, cell monolayers were wounded by scratching the surface of the 12-well plate as uniformly as possible with a pipette tip. The scratched wells were washed with PBS to remove detached debris after scratching. Then, fresh RPMI1640 medium or medium containing indicated inhibitors were added to the scratched wells. The cells were then incubated for 15 h. The initial wounding and movement of the cells in the scratched area were photographically monitored and imaged using an Olympus microscope coupled with a digital imaging camera system at 0 h and 15 h.

### *In vitro* migration assay

Migration of cells was assessed in a 24-well plate Transwell system (Costar, Corning, USA) as described previously^52^. SKOV3 stable cells were seeded at a density of 1 × 10^4^, and HEC-251 stable cells were seeded at a density of 2 × 10^4^ per well onto 8 μm Transwell inserts. Each insert was filled with 100 μl of cells with serum free RPMI1640, and the lower chamber was filled with 500 μl RPMI1640 containing 10% FBS. The cells were incubated for 48 h. Pictures of the membrane were taken in 5 random fields per chamber, and the total number of migrated cells was counted. Experiments were performed in triplicate.

### Propidium Iodide staining and Flow cytometry

SKOV3 and HEC-251 cells (1 × 10^6^/100 mm dish) were harvested and fixed overnight with ice-cold 70% ethanol. The samples were analyzed as previously described^53^.

### Immunoprecipitation and PP2A phosphatase activity assay

FLAG-tagged PPP2R1A constructs were transfected into HEK293 cells using PEI. After 48 h, the cells were lysed in IP buffer as previously described^54^. For the PP2A activity assay, phosphates were removed using phosphate removal columns (MPR020, Profoldin, MA, USA). Then, cell lysates were incubated for 2 h at 4 °C with 1 μg of antibody against PP2Ac, followed by an additional 2 h of incubation with protein-G-agarose beads (Roche). After three washes, immunoprecipitates were used in a phosphatase reaction according to the manufacturer’s instructions (Promega).

### Xenografts

All procedures were approved by the Institutional Animal Care and Use Committee of Sookmyung Women’s University, Seoul, South Korea. The methods were carried out in accordance with the approved guidelines. The mice were injected with 100 μl (50% Matrigel/PBS) SKOV3 cells at a concentration of 2 × 10^6^ and HEC-251 cells at a concentration of 6 × 10^6^ cells/100 μl PBS subcutaneously into the flanks of 7-week-old athymic nu/nu mice (NCI). Three female mice were used in each group. Tumor growth was monitored by measuring the tumor volumes (length × width^2^ × 0.52) once a week with calipers. Mice were euthanized and tumors were harvested to measure tumor weight.

## Additional Information

**How to cite this article**: Jeong, A. L. *et al.* Patient derived mutation W257G of *PPP2R1A* enhances cancer cell migration through SRC-JNK-c-Jun pathway. *Sci. Rep.* **6**, 27391; doi: 10.1038/srep27391 (2016).

## Figures and Tables

**Table 1:** Expression of *PPP2R1A* in human normal and cancer tissues (Data are taken from the cancer microarray database Oncomine).

Class I versus Class II (Sample Number)		Correlation (up/down)*	Fold Change	p-value	Reference
Analysis type: Normal versus Cancer					
Ovarian Surface Epithelium (10)	High-grade Ovarian Serous Carcinoma (185)	↑	1.680	4.73E-6	33
Ovarian Surface Epithelium (5)	Ovarian Clear Cell Adenocarcinoma (7)	↑	1.233	0.036	34
No value (8)	Ovarian Serous Cystadenocarcinoma (586)	↓	−1.369	1.000	TCGA Data
Myometrium (27)	Uterine Corpus Leiomyoma (50)	↑	1.160	0.033	35
Skin (7)	Cutaneous Melanoma (45)	↑	4.527	1.34E-5	36
Plasma Cell (5)	Multiple Myeloma (133)	↑	1.419	6.95E-4	37
No value (10)	Lung Adenocarcinoma (86)	↑	1.312	7.37E-7	32
Breast (144)	Invasive Lobular Breast Carcinoma (148)	↑	1.525	1.03E-45	39
Breast (144)	Invasive Ductal Breast Carcinoma (1,556)	↑	1.567	1.5E-65	39
Bladder (9)	Superficial Bladder Cancer (28)	↑	1.751	9.40E-8	40
Bladder (9)	Infiltrating Bladder Urothelial Carcinoma (13)	↑	1.670	1.01E-5	40
Bone Marrow (22)	Smoldering Myeloma (12)	↑	2.465	3.49E-6	38

^*^Represents increased expression in class 2 compared to that in class 1.

**Table 2:** Mutant types of *PPP2R1A* in various cancer studies.

Cancer Study	A.A. change^×^	Type	Copy^#^	COSMIC^*^
Lung squamous cell carcinoma (TCGA)Uterine Corpus Endometrioid Carcinoma (TCGA)	R144H/C	Missense	Diploid	H 1/C 1
Uterine Carcinoma (TCGA)Uterine Corpus Endometrioid Carcinoma (TCGA)	P179R/L	Missense	Deepdel^+^ 1Shallowdel° 5Diploid 2Gain 4	L 5/R 26
Breast Invasive Carcinoma (TCGA)Colorectal adenocarcinoma (TCGA)Lung Adenocarcinoma (Broad)Ovarian Serous Cystadenocarcinoma (TCGA)Uterine Carcinosarcoma (TCGA)Uterine Corpus Endometrial Carcinoma (TCGA)	R183W	Missense	Shallowdel 2Diploid 7Gain 1	29
Uterine Carcinosarcoma (TCGA)Uterine Corpus Endometrial Carcinoma (TCGA)	S256Y/F	Missense	ShallowDel 1Diploid 3Gain 2	Y 5/F 12
Kidney Renal Clear Cell Carcinoma (TCGA)Ovarian Serous Cystadenocarcinoma (TCGA)	W257*/C/G	NonsenseMissense	Diploid 2Gain 2	C 6/G 3
Colorectal Adenocarcinoma (TCGA)Lung Adenocarcinoma (TCGA)Uterine Corpus Endometrial Carcinoma (TCGA)	R258H/C	Missense	Diploid 4Gain 2	H 9/C 2

^×^Amino acid change.

^*^Overlapping mutations in COSMIC (count).

^+^Deep deletion.

^°^Shallow deletion.

**Table 3:** Mutation sites and mutation rates of *PPP2R1A* in various cancer studies.

Cancer type	*PPP2R1A*mutation rate*	Mutation site	Count	Ref.
Type I Ovarian carcinomas(clear-cell, low-grade endometrioid and serous carcinomas)	9.1% (10/110)	P179R183S256W257	11231	10
Type II Ovarian carcinomas (high-grade serous carcinomas)	0% (0/71)			
Endometrial endometrioid carcinomas	6.7% (2/30)			
Endometrial serous carcinomas	19.2% (5./26)			
Type I Ovarian carcinomas(clear-cell, low-grade endometrioid and serous carcinomas)	7.06% (7/102)	P179R183S256W257	10783	55
Type II Ovarian carcinomas(high-grade serous carcinomas)	0% (0/50)			
Endometrial endometrioid carcinomas	5% (3/60)			
Endometrial serous carcinomas	40.8% (20/49)			
Endometrial endometrioid carcinomas	7.1% (22/306)	P179R183S256W257	151172	56
Endometrial serous carcinomas	43.2% (16/37)			
Endometrial serous carcinomas	25% (13/52)	P179R183S256W257	5034	7

^*^Mutation rate (count/case).
